# Looking back to the recent past: the influence of national health policies and vaccination during the COVID-19 pandemic on maternal health

**DOI:** 10.61622/rbgo/2025rbgoedt11

**Published:** 2025-11-18

**Authors:** Renato Teixeira Souza, Maria Laura Costa, Jessica Lynn Schue, Emily Miller, Rupali Jayant Limaye, Jose Guilherme Cecatti

**Affiliations:** 1 Universidade Estadual de Campinas Department of Obstetrics and Gynaecology Campinas SP Brazil Department of Obstetrics and Gynaecology, Universidade Estadual de Campinas, Campinas, SP, Brazil.; 2 Faculdade de Medicina de Jundiaí Jundiaí SP Brazil Faculdade de Medicina de Jundiaí, Jundiaí, SP, Brazil.; 3 Johns Hopkins University Bloomberg School of Public Health Department of International Health Baltimore USA Department of International Health, Bloomberg School of Public Health, Johns Hopkins University, Baltimore, USA.

The COVID-19 pandemic posed profound challenges across healthcare systems globally. In Brazil, its impact has been particularly severe in maternal health, reversing years of progress, exposing long-standing socioeconomic and regional inequities. For instance, before the pandemic, Brazil had been decreasing its maternal mortality ratio (MMR), though unevenly across regions; however, with the advent of COVID-19, maternal mortality rose sharply, reaching double the ratio in some analyses.^(1)^ This increase was driven both by direct causes (COVID-19 infection in pregnant or postpartum women) and indirect causes (interruptions in care, delays, reduced access to prenatal and obstetric services). The rise in MMR was not uniform. The North, Midwest, and some parts of the Northeast saw larger increases. Also, more deaths occurred in the postpartum period. Socioeconomic factors played a role: women with lower education, black women, and those in more remote or under-resourced regions suffered disproportionately.

Under this scenario, prenatal care was disrupted, with a reduced number of consultations, some postponed or cancelled, and longer delays in recognising or treating complications. Hospitals and maternity units diverted resources to COVID-19, sometimes resulting in fewer beds, ICU capacity constraints, delays in testing, or reduced capacity to monitor obstetric complications.^(2)^ In addition, the spread of misinformation and a lack of clear and evidence-based orientations from the Ministry of Health and other governmental agencies regarding the best procedures and management dealing with COVID-19 at the population level and specifically during pregnancy. Furthermore, criticism and negationism also contributed to the chaos in adequately approaching the problem in the health system and with the population.

After what could be called a never-before-seen effort, several types of vaccines against COVID-19 were developed simultaneously and were available to be commercially used one year after the onset of the pandemic. They were soon proven to help prevent severe disease, hospitalisation, ICU admission, and death in pregnant and postpartum women. Therefore, reducing the burden of COVID-19 in this group contributed directly to lowering maternal mortality after their introduction on a large scale. Additionally, the vaccine also helps decrease indirect harms: fewer infections in pregnant women means less strain on the health service, less risk of overwhelming ICUs, and better availability for obstetric emergencies.

Vaccination led to an important reduction in maternal mortality in Brazil; however, regional comparisons still reveal huge disparities with much higher vaccination coverage among pregnant/puerperal women in the Southeast and South compared to very low levels in other states from the North and Northeast regions.^(3)^

Initially, pregnant and postpartum women were not always prioritised in vaccine rollouts globally or within Brazil. The delay in recognising their higher risk may have contributed to the excess mortality that occurred. Over time, as evidence became available, public health bodies in Brazil developed guidelines recommending vaccination during pregnancy/postpartum, and working groups formed to address adequate care. The Ministry of Health in Brazil issued normative guidance to orient the care of pregnant/puerperal women, and working groups of obstetricians/gynaecologists coordinated efforts to share protocols, case reviews, and training.

In fact, there were multiple scenarios, also with a multitude of responses to dealing with obstetric care during the pandemic. Testing policies were inconsistent; while some rare centres did universal screening near delivery, many others only tested moderate or severe cases. Some units increased staffing of shifted workloads, others had to suspend annual leaves, and redeploy staff. Nevertheless, the pandemic also exhibited the scientific community´s rapid mobilisation. A positive learning from this experience was how scientists and researchers behaved in the face of the pandemic, trying to have a comprehensive understanding of the disease and its epidemiology, biology, ways of transmission and ways of avoiding it, identifying risk factors for acquiring or getting worse with the infection, proposing measures for population control of the spread and working in laboratories for deciphering its molecular characteristics and immunogenicity for the vaccines, besides exploring why hesitancy currently still stands as a key issue.

As vaccines became available in Brazil, pregnant and postpartum women were eventually included in priority groups, but not from the very beginning. Vaccination coverage among pregnant/postpartum women varied greatly by state/region. Some states achieved high uptake, others very low. The national Immunisation Program (PNI - Programa Nacional de Imunizações) is historically strong in Brazil, and some of that infrastructure was leveraged. However, the pandemic introduced many new logistical, behavioural, and policy challenges.

Some barriers and challenges arose from distrust of vaccines, spread of misinformation and fake news that have been significant issues. Political polarisation worsened the situation, with opinions about vaccines sometimes aligned with political lines, undermining unified messaging, leading to vaccine hesitancy, which has never been a characteristic of the Brazilian population before. While some geographic and social disparities still exist, coordination challenges need to be faced through alignment among federal, state and municipal authorities in the implementation of opportune measures to deal with COVID-19 or even future new epidemics.

Therefore, if it is possible to make some recommendations, they should include: to ensure timely vaccination of all pregnant and postpartum women, including boosters, using outreach in prenatal clinics; to address vaccine hesitancy with clear, consistent, culturally appropriate messaging, engaging trusted community actors, health professionals, and ensuring transparency; to improve prenatal, childbirth, and postpartum care infrastructure, increasing ICU and emergency obstetric capacity especially in vulnerable regions; to expand testing and screening, with universal screening protocols in maternity wards where feasible; to support the health workforce: mental health support, training, and adequate protection; to monitor and evaluate continuously maternal morbidity/mortality, vaccine effectiveness in pregnancy, and indirect effects, to adapt policies.

The COVID-19 pandemic has shown how maternal health is especially vulnerable in the face of a large infectious disease outbreak, particularly when health systems are stressed, inequities do exist, and preventive tools (like vaccines) are not deployed early and equitably. Brazil´s experience offers both cautionary lessons and examples of what can work. Investments that strongly support health system capacity, ensure equitable vaccine access, reduce regional disparities, and build trust will not just help in this pandemic but strengthen preparedness for future health emergencies.
